# The Rapid Phenotypic Susceptibility Testing in Real-Life Experience: How the MIC Values Impact on Sepsis Fast Diagnostic Workflow

**DOI:** 10.3390/diagnostics14010056

**Published:** 2023-12-26

**Authors:** Giuseppe Migliorisi, Maddalena Calvo, Antonina Collura, Francesca Di Bernardo, Marianna Perez, Guido Scalia, Stefania Stefani

**Affiliations:** 1 U.O.C. Laboratory Analysis A.O.U. “Policlinico—San Marco”, Via Santa Sofia 78, 95123 Catania, Italy; maddalenacalvo@gmail.com (M.C.); stefania.stefani@unict.it (S.S.); 2 U.O.C. Clinical Microbiology, “Civico-Di Cristina-Benfratelli” Hospital, Piazza Nicola Leotta 4, 90127 Palermo, Italy; 3 Department of Biomedical and Biotechnological Sciences, University of Catania, Via Santa Sofia 97, 95123 Catania, Italy

**Keywords:** sepsis, MIC value, rapid phenotypic AST

## Abstract

The MIC value definition faithfully reflects antimicrobial sensitivity, profoundly impacting the infection's clinical outcome. Our study aimed to evaluate the Accelerate Pheno^TM^ System in defining the importance of fast phenotypic susceptibility data. A number of 270 monomicrobial samples simultaneously underwent standard procedures and fast protocols after a contemporary Gram stain. Finally, we provided Turn-around Time (TAT) and statistical evaluations. The fast technology required a medium value of 7 h to complete ID and AST profiles. Although there were some spectrum limitations, it revealed an optimal success rate in microbial identification directly from positive blood cultures. The Gram-negative AST reached a 98.9% agreement between the Accelerate Pheno™ System and the standard method. In addition, the Gram-positive AST gathered a 98.7% agreement comparing the same systems. The chance to rapidly provide precise MIC values is one of the last frontiers in clinical microbiology, especially in high-prevalence antimicrobial resistance areas.

## 1. Introduction

The minimum inhibitory concentration (MIC) value determination is currently the best tool for guiding clinical decisions about therapeutic options after the application of the proper in-force guidelines. The MIC value definition faithfully reflects antimicrobial susceptibility, profoundly impacting the infection's clinical outcome [1,2]. Despite the methodological limitations of the broth microdilution method (BMD), this procedure is currently considered the most reliable technique for AST definition [3]. Noticeably severe systemic infections demand a rapid diagnosis and an early MIC parameter definition due to their time-sensitive nature. Among these infections, sepsis is a critical, life-threatening condition that requires shorter diagnostic periods to prevent severe complications [4].

According to this urgency, innovative diagnostic tools have been proposed to provide identification (ID) and antimicrobial susceptibility testing (AST) information in a shortened period [4,5,6]. The Accelerate Pheno^TM^ System panel is an automated system that performs rapid ID and AST directly from positive blood culture samples, confirming high specificity and sensitivity rates [7]. The method combines fluorescent in situ hybridizations for ID and morphokinetic cellular analysis with timelapse microscopy for AST. The main advantage is the possibility of gathering a partial and informative microbiological report within 7 h. Comprehensively, this technology has actualized a reliable and rapid phenotypic method for assessing susceptibility test results to the most valuable antimicrobials in the case of systemic infection. This feature has developed into a crucial point for targeted antibiotic therapy optimization [8].

Our study, performed in two clinical microbiological centers in Southern Italy, was planned to provide an algorithm that aims to demonstrate the effectiveness of the Accelerate Pheno^TM^ System in the sepsis diagnostic workflow. The definitive rationale for performing such an experimental protocol is significantly related to our country’s epidemiological features concerning isolated microbial species and detected antimicrobial resistance genes. As a matter of fact, an average value of 47.2% of carbapenem-resistant species and 57.1% of ESBL-producing species were recorded from the Civico-Di Cristina-Benfratelli Hospital of Palermo and the Policlinico-San Marco Hospital of Catania during the last years [9]. Moreover, 61.9% of multi-drug-resistant isolates were included in the *Klebsiella pneumoniae* species (among 536 detections), while 36.2% were *Acinetobacter baumannii* (among 206 detections). Approximately 21.9% of isolated strains (among 336 detections) were recorded as methicillin-resistant *Staphylococcus aureus* (MRSA). Finally, 14.3% of vancomycin-resistant enterococci (VRE) were noticed (among 179 detections) [9]. The alarming spread of antimicrobial resistance in our specific area led us to hypothesize a possible rapid sepsis diagnostic workflow for correct and prompt patient management.

## 2. Materials and Methods

An experimental design was planned from January to December 2022, involving the Civico-Di Cristina-Benfratelli Hospital of Palermo and the Policlinico-San Marco Hospital of Catania. This study only included biological samples and did not require any intervention on human beings. The enrolled samples were part of the conventional diagnostic workflow, whose guidelines included a blood culture sample in the case of a sepsis suspicion. On that premise, this study did not involve any supplementary samples or the collection of patients’ clinical data.

Both involved centers have standardized the same protocol with reference to inclusion or exclusion criteria and analytical stages. Globally, 270 blood samples were collected from patients recovered in intensive care, internal medicine, hematology, surgery, and emergency units. Patients from these wards have been considered critical subjects, and clinicians have always requested a fast-track protocol in the case of blood sample positivity.

All the processed samples flagged as positive by the BACTEC^TM^ FX System underwent standard and rapid protocols. Specifically, the standard procedure consisted of a Gram-stain, whose results were timely transferred to clinicians. In addition, the Gram stain was used as a parameter to exclude polymicrobial and yeast samples from the rapid protocol.

Then, the same samples underwent culture-based methods according to international guidelines [10]. The identification step was provided through the MALDI Biotyper^®^ System (Bruker) for all the cultured isolates from the included samples. In addition, BD Phoenix^TM^ (NMIC-474 or PMIC-96 panels) was used to provide automated AST from grown bacterial colonies within 24 h. The Kirby–Bauer and Gradient test techniques were applied for uncommon species (such as *Aeromonas hydrophila*, *Bacteroides fragilis*, *Pseudomonad stutzeri*, *Stenotrophomonas maltophilia*, and *Corynebacterium striatum*), whose susceptibility profiles are not available on the automated systems.

The rapid protocol involved the Accelerate Pheno™ System simultaneously with the standard procedures hours earlier. The system was used only for samples whose positivity had been flagged no more than 8 h earlier. This rapid technology provided ID within 1 h and 30 min and a preliminary susceptibility profile within 7 h. All the conventional and rapid MIC results have been categorized according to the European Committee on Antimicrobial Susceptibility Testing (EUCAST) guidelines [11].

**Figure 1:**
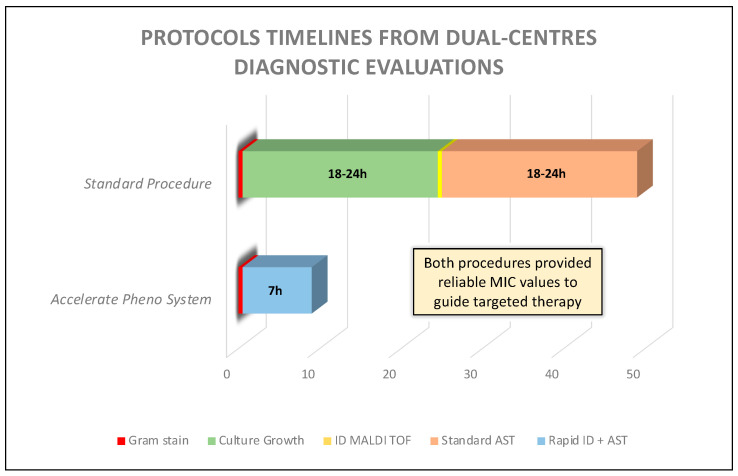
Graphical abstract to illustrate TAT analysis comparing the standard procedure to the Accelerate Pheno^TM^ System (Accelerate Diagnostics, Denver, CO, USA).

This system required 2 mL of positive blood to execute automated fluorescence in situ hybridization for ID and morphokinetic cellular analysis for AST. The Accelerate Pheno™ results were integrated with smart reporting and directly forwarded to microbiologists and clinicians through an online communication system. Figure 1 illustrates the sepsis diagnostic workflow planned for this study.

Additionally, a molecular diagnostic confirmation was planned for those antimicrobial resistances that were epidemiologically relevant in our geographical area. Particularly, carbapenem-resistance, methicillin-resistance, and vancomycin-resistance genes were detected through the Cepheid^®^ GeneXpert System (Cepheid, Sunnyvale, CA, USA). Molecular results were added to the phenotypic susceptibility report.

A comparison between the standard protocol results and the rapid protocol reports has been produced. The turn-around time (TAT) was calculated for each protocol. ID and/or AST discrepancies were noticed compared to the standard method. MIC value discrepancies were further investigated through the EUCAST broth microdilution, which is recognized as the universal standard method. Successful ID and AST were defined as ID and AST matching the standardized results.

Valuable AST results were defined as profiles where MIC values differed no farther than a 1-fold dilution regarding the standard method, not affecting the EUCAST categorization. The essential agreement (EA) and the categorical agreement (CA) were calculated according to current guidelines [12].

## 3. Results

### 3.1. Sample Size and Identification Results

A total of 515 blood culture samples were collected among the two hospital centers. Specifically, 270 samples revealed a monomicrobial aetiology after an extemporary Gram stain, thus they were included in the experimental protocol. Among these monomicrobial samples, 215 blood cultures were from adult patients and 55 from pediatric patients. Additionally, 191 samples revealed Gram-negative bacteria, while 79 showed Gram-positive bacteria. Finally, 245 blood samples were excluded from the rapid protocol due to the presence of yeast (121) or polymicrobial aetiology (124).

Notably, 119 isolates belonged to the *Enterobacterales* (66 *Klebsiella pneumoniae* subspecies *pneumoniae*, 40 *Escherichia coli*, 4 *Proteus mirabilis*, 3 *Klebsiella aerogenes*, 2 *Klebsiella oxytoca*, 2 *Citrobacter koseri*, 1 *Morganella morganii*, and 1 *Serratia marcescens* strain). Additionally, 30 *Klebsiella pneumoniae* isolates emerged as KPC-producing.

Obligate anaerobic microorganisms, such as *Bacteroides fragilis* (3), were also detected. Furthermore, uncommon species were noticed, such as *Aeromonas hydrophila* (2) and *Pseudomonas stutzeri* (1). Moreover, non-fermentative Gram-negative rods were identified as *Acinetobacter baumannii* (43), *Pseudomonas aeruginosa* (13), and *Stenotrophomonas maltophilia* (10).

In relation to the Gram-positive bacteria, 44 isolates belonged to the *Staphylococcus* genus (34 *Staphylococcus aureus*, 8 *Staphylococcus epidermidis*, and 2 *Staphylococcus hominis*). Furthermore, 11 *Staphylococcus aureus* tested positive for cefoxitin screening, resulting in MRSA.

Otherwise, 28 strains were identified within the *Enterococcus* genus (19 *Enterococcus faecium* and 9 *Enterococcus faecalis*). In addition, 5 *Enterococcus faecium* resulted in VRE. Additionally, 4 isolates belonged to the *Streptococcus* genus (1 *Streptococcus pneumoniae*, 1 *Streptococcus agalactiae*, and 2 *Streptococcus mitis*). Finally, 3 isolates were identified as *Corynebacterium striatum*.

Table 1 summarizes all the Gram-negative and Gram-positive bacterial species identified from enrolled positive blood cultures. The time to positivity was observed to be different for the two groups. Specifically, Gram-negatives showed an average time to blood-culture positivity of 8 h, though Gram-positives reported 15 h. 

### 3.2. Fast Protocol Limitations

First, the Accelerate Pheno™ System cannot process a blood sample flagged as positive more than 8 h earlier. Among our identified species, we noticed the presence of 20 off-panel microorganisms. Specifically, 17 Gram-negative bacteria (10 *Stenotrophomonas maltophilia*, 3 *Bacteroides fragilis*, 2 *Aeromonas hydrofila*, 1 *Morganella morganii*, and 1 *Pseudomonas stutzeri*) and 3 *Corynebacterium striatum* were observed. These species were not included in the Accelerate Pheno™ System identification panel, which could not perform a rapid AST. Unfortunately, these limitations resulted in off-panel results compared to the standard procedure.

Furthermore, *Streptococcus* spp. was defective due to the inability of the Accelerate Pheno™ System to provide an AST result, although the panel furnishes a correct genus identification. In addition, the Accelerate Pheno™ System is able to identify only the genus level of the following microorganisms: *Klebsiella* spp., *Citrobacter* spp., and *Proteus* spp., whose AST profiles are correctly completed by the system. The impossibility of identifying the above-mentioned microorganisms at a species level led to agreement values that were not specifically distinguished between species or genus identifications.

All the off-panel microorganisms and the genus-level identified strains were processed through the conventional protocol. Indeed, the MALDI Biotyper^®^ System (Bruker Daltonics, Bremen, Germany) provided complete species identification for all the above-mentioned microorganisms.

### 3.3. Agreement Rates

An agreement percentage of the rapid technology compared to the standard method was calculated, excluding the off-panel microorganisms. Specifically, the Accelerate Pheno™ System revealed a success rate of 100% in Gram-positive and Gram-negative identifications.

Furthermore, the EA in AST was analyzed between the Accelerate Pheno™ System and the standard method. Approximately 98.9% of EA (1.1% of discrepancies related to two discrepant strains) was achieved in Gram-negative AST between the Accelerate Pheno™ System and the standard method. The CA showed the same value (98.9%), revealing two strains (1.1%) with a MIC value difference of more than one dilution between the two compared methods. Precisely, two discrepancies were recorded about colistin for *K. pneumoniae* due to a resistant MIC value (≥8 mg/L) from the Accelerate Pheno™ System and a susceptible MIC value ≤ 0.5 mg/L from the BD Phoenix^TM^ NMIC-474 panel. According to current guidelines, these two disagreements were initially classified as major errors [10]. Despite this assumption, we further investigated the discrepancies through EUCAST broth microdilution, which revealed a resistant MIC value equal to 4 mg/L.

In addition, an EA of 98.7% (1.3% of discrepancies related to only 1 strain) was gathered from Gram-positive AST between the two systems. The CA revealed the same value (98.7%), due to a linezolid discrepancy for *E. faecalis*. Specifically, the Accelerate Pheno™ System detected a resistant MIC value > 4 mg/L, while the BD Phoenix^TM^ revealed a susceptibility MIC value equal to 2 mg/L.

According to the above-mentioned guidelines, the discrepancy was classified as a major error but was further investigated through EUCAST broth microdilution, which revealed a MIC value of 2 mg/L. Very major errors or minor errors were not detected.

Finally, as regards pediatric samples, the success rate for this type of sample reached 96.4% (3.6% of off-panel microorganisms).

### 3.4. Turn-Around Time Analysis

**Figure 2:**
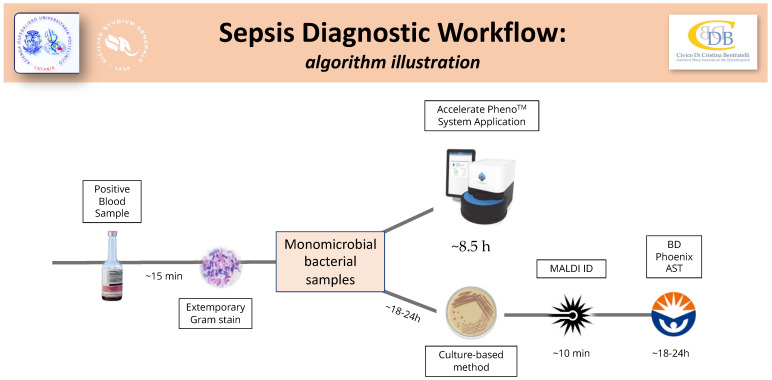
Schematic illustration of the Sepsis Diagnostic Workflow for positive blood cultures. Simultaneous standards and fast procedures were applied to the collected positive blood cultures. Only monomicrobial bacterial samples underwent the fast protocol.

Standard procedures required 48–72 h to provide a definitive report because of the mandatory overnight incubation period (18–24 h) with the antimicrobial susceptibility test (15–18 h). Eventual additional susceptibility tests could require a further 18–24 h. On the contrary, the Accelerate Pheno™ System produced an ID and AST intermediate report in 7 h, confirming a significant time-saving in sepsis diagnostic procedures. Figure 2 summarizes the two protocols’ timelines, describing TAT differences between standard and rapid methods.

## 4. Discussion

The current guidelines for sepsis management state the urgent need to administer targeted antimicrobial therapy. Therefore, it is essential to rapidly provide ID and AST to apply an appropriate stewardship strategy [13]. Moreover, the chance to include the definition of precise MIC values within a fast technology has become one of the last frontiers in clinical microbiology. Several scientific publications highlight the impact of the MIC value on therapeutic assessment and the patient’s outcome. Additionally, the variable expression level of some resistance patterns may cause MIC variability. The MIC values alone are not enough to establish a correct therapy, but they currently represent the most reliable and available parameter to reveal the efficacy of antimicrobial molecules. As a result, the MIC value is the key to enriching the susceptibility profile with pharmacological parameters. Those pharmacokinetic and pharmacodynamic (PK/PD) properties are sometimes crucial to predicting a patient’s outcome during severe infections, and they could not be defined without an initial MIC value [14]. Indeed, the MIC value appears to be one of the most important topics during clinician and laboratory personnel evaluations of critical patients. Among the antimicrobial drug selection criteria, MIC values still represent significant data.

Considerable progress has recently been made in antimicrobial treatments and sepsis diagnostic technologies, allowing microbiologists to identify pathogenic microorganisms and their susceptibility profiles ahead of time [7]. Among available technologies, the Accelerate Pheno^TM^ System captured our attention due to the possibility of providing rapid ID and phenotypic AST directly from positive blood cultures. It has also fascinated us owing to the ease and rapidity of preparing the panels from the blood samples. In addition, the Accelerate Pheno^TM^ System is the only technology currently available to provide rapid ID and phenotypic AST directly from blood samples for diagnostic use.

During the conceptualization stages of this study, we evaluated the impact the Accelerate Pheno^TM^ System could have on our healthcare setting. Remarkably, we operate in a high-prevalence antimicrobial resistance area where it is essential to adopt urgent measures for managing critical patients. Our data recorded significant percentages of multi-drug-resistant microorganisms (MRSA, VRE, and CRE). These pathogens easily spread in our hospitals because of copious recoveries and frequent patient relocations. Moreover, some strains often express hypervirulence and persistence among critical patients, complicating eradication and treatment. The most reliable way to prevent alert microorganism spread and persistence is to apply urgent diagnostic measures after the systemic infection suspicion. The rapid responses may also become the start of efficient patient coordination in the case of multi-drug resistance etiologies, which require infection control measures. On that premise, the wide diffusion of multi-drug-resistant microorganisms from most investigated clinical wards has been reported.

Although monomicrobial infections still hold the record in sepsis aetiology, our investigations also reported an extensive case series of polymicrobial blood samples. We decided to briefly point out the low sensitivity of the Accelerate Pheno^TM^ System in the case of polymicrobial specimens, which were not included in the fast protocol. In our experience, the system is neither often able to distinguish different microorganisms from the same samples nor provide their specific AST profile. For these reasons, the decision to exclude all the polymicrobial specimens from the fast protocol evaluations has been settled. However, polymicrobial episodes always deserve some consideration. The simultaneous presence of more than one pathogen at a sterile site requires careful interpretation according to the characteristics of the pathogenetic process. Contamination processes and rates should be considered during the clinical evaluation of polymicrobial reports.

Furthermore, although bacterial aetiology still represents the first sepsis cause, fungal isolates are often involved in severe infections in our hospital settings. Hence, we faced a particular epidemiological condition in which different yeast species are diffused among critical wards. In our opinion, the Accelerate Pheno^TM^ System was not the best option to investigate fungal aetiology due to a restricted species identification spectrum (*Candida albicans* and *Candida glabrata*) and the inability to define any antifungal susceptibility profile.

The Accelerate Pheno^TM^ System helped define the sepsis aetiological agent of most analyzed samples. These cases were promptly managed thanks to the rapid communication of AST results to clinicians. This communication was possible through the smart reporting option, which allowed the mailing forwarding of ID and AST results immediately after the process was completed. Clinicians early received the rapid result, considering its data as preliminary and using them to guide the antimicrobial treatment. The possibility of rapidly furnishing a precise MIC value guided the targeted therapy for better clinical practice.

Our evaluations confirm that the system benefits emanate from excellent sensitivity and specificity rates, including molecular probes within the identification process. ID results revealed an optimal agreement level (100%) compared with the ultimate generation of mass-spectrometric techniques. In our critical hospital settings regarding antimicrobial resistance spread, smart reporting through online systems has profoundly revolutionized the clinical management of septic patients. Obviously, the system rejection of blood samples flagged as positive more than 8 h earlier leads to the necessary logistical organization of hospital personnel. Microbiologists and clinicians should always be available to receive and apply rapid protocol results, considering as mandatory a minimum of a 12 h guard setting for every day of the week. Furthermore, the Pheno Test BC kit is simple to use, and any laboratory personnel can be trained to initiate the panel as soon as the blood samples turn positive.

Unfortunately, some microorganisms resulted in non-applicable identification due to a restricted species spectrum. In those cases, the technology measured significant microbial growth without the chance to assign a defined genus or species identification.

Regrettably, the limited identification panel had compromised the timesaving of some critical cases, which were investigable only through culture-based methods. However, only 7.4% of processed samples faced this difficulty, highlighting the high success percentage and the uncommon aetiologies that rarely occur among sepsis episodes.

Our study also described a few cases where the Accelerate Pheno^TM^ System identification was not followed by the definition of an AST profile. These technical panel limitations allowed the report of only partial information, which required waiting for the culture-based results. The limitations encourage further studies about the extension of the rapid panels to optimize blood culture analysis within fast protocols. Despite the few limited episodes, the system performed an AST in most cases during our evaluations. In addition, the AST agreement rate with the conventional method was high for Gram-negatives (98.9%) and Gram-positives (98.7%). The successful rate is attributable to the innovative AST definition process, which combines time-lapse microscopy with morphokinetic analysis, promising ideal levels of reliability.

Interestingly, the system does not report AST results in the case of insufficient blood volume or inadequate microbial growth in the positive blood samples. This built-in quality technology has established quantitative limits to complete all the processes based upon dynamic dilution, an automated feature of the PhenoTest BC kit. Specifically, a quantity lower than 10 microbial cells does not allow the production of either ID or AST. On the other hand, a number larger than 130 microbial cells does not allow the definition of the AST, although ID is regularly completed. However, similar cases did not occur during our investigations, encouraging the accuracy of the technology used.

Notably, a few discrepancies between the fast system and the standard protocol were reported regarding the MIC value definitions of linezolid for *E. faecalis* and colistin for *K. pneumoniae*. Those cases were investigated through EUCAST broth microdilution, and they revealed different conclusions.

As regards linezolid, EUCAST broth microdilution confirmed the BD Phoenix^TM^ value, highlighting the importance of a critical interpretation of resistance MIC values. In connection with colistin, the EUCAST microdilution endorsed the Accelerate Pheno^TM^ System result, confirming the incompetence of the traditional automated systems in defining MIC values for “difficult” molecules. As regards official EUCAST recommendations, colistin susceptibility assays should be categorized only after the application of a broth microdilution. Therefore, conventional automated systems are not able to provide a viable result.

In this discussion, we would like to focus on pediatric samples, which are often affected by low blood volume, compromising the effectiveness of rapid tests. Low sensitivity rates and difficulties in obtaining a suitable sample often pose challenges to sepsis diagnosis among pediatric samples. We recognized the importance of correctly responding to such particular microbiological requests through rapid workflows along with conventional methods. According to our gathered data, the success rate for this type of sample reached 96.4% (3.6% of off-panel microorganisms). In conclusion, the Accelerate Pheno^TM^ System optimized the sepsis diagnostic workflow among pediatric wards, which perfectly matches the definition of urgent procedures.

## 5. Conclusions

Although all diagnostic tests have certain limitations, our algorithm application was successful for most critically ill patients because of the high agreement rates with conventional protocols [15]. Noticeably, Europe is facing significant difficulties with antimicrobial resistance diffusion, and Italy holds a 26.7% antimicrobial resistance rate [16,17,18,19], supporting the urgency to cleverly integrate the ultimate advanced diagnostic tools in severe infection diagnosis. Several technologies are currently available for rapidly providing microbiological data [20,21,22,23,24], enhancing the importance of providing precise and specific results early. However, these technologies should be integrated into a well-defined workflow because of their different features, which require careful interpretation. For instance, molecular systems for detecting resistance genes are easy to use and rapid [25,26,27,28,29], but they do not procure complete information to determine the correct antimicrobial treatment. However, these methods are well integrated into urgent diagnostic procedures based on different biological samples. Their results should be considered an added tool to phenotypic susceptibility data, whose essential MIC values may be partially supplemented by resistance gene detection. The need to integrate different diagnostic tools into the same workflow highlights the essential role of the clinical microbiology laboratory in severe infection diagnosis [30]. The Accelerate Pheno^TM^ System is an ideal diagnostic option for severe infections, providing MIC values at brief intervals. Among all the microbiological data, the defined MIC values are the fundamental tool to really impact patients’ clinical and therapeutic management. A fundamental purpose is to explain how competence and knowledge about pharmacological parameters and patients’ clinical conditions may be crucial in sepsis management. The ultimate aim of our study is to encourage further clinical evaluations about the cost-effectiveness of such rapid systems, which really have the potential to modify clinicians’ decisional processes and critical patient outcomes.

## Figures and Tables

**Table 1:** Species identified in processed blood samples.

Gram-Negative Bacteria	Isolates (191)
*Acinetobacter baumannii*	43
*Aeromonas hydrophila*	2
*Bacteroides fragilis*	3
*Citrobacter koseri*	2
*Escherichia coli*	40
*Klebsiella aerogenes*	3
*Klebsiella oxytoca*	2
*Klebsiella pneumoniae* subspecies *pneumoniae*	66
*Morganella morganii*	1
*Proteus mirabilis*	4
*Pseudomonas aeruginosa*	13
*Pseudomonas stutzeri*	1
*Serratia marcescens*	1
*Stenotrophomonas maltophilia*	10
**Gram-positive bacteria**	**Isolates (79)**
*Corynebacterium striatum*	3
*Enterococcus faecalis*	9
*Enterococcus faecium*	19
*Staphylococcus aureus*	34
*Staphylococcus epidermidis*	8
*Staphylococcus hominis*	2
*Streptococcus agalactiae*	1
*Streptococcus mitis*	2
*Streptococcus pneumoniae*	1

## Data Availability

All data generated or analyzed during this study are included in this article.
